# Outcome of neurological early rehabilitation patients carrying multi-drug resistant bacteria: results from a German multi-center study

**DOI:** 10.1186/s12883-017-0833-2

**Published:** 2017-03-20

**Authors:** J. D. Rollnik, M. Bertram, C. Bucka, M. Hartwich, M. Jöbges, G. Ketter, B. Leineweber, M. Mertl-Rötzer, D. A. Nowak, T. Platz, K. Scheidtmann, R. Thomas, F. von Rosen, C. W. Wallesch, H. Woldag, P. Peschel, J. Mehrholz, M. Pohl

**Affiliations:** 1 Institute for Neurorehabilitation Research (InFo), BDH-Klinik Hessisch Oldendorf, Hannover Medical School (MHH), Greitstr. 18-28, 31840 Hess. Oldendorf, Germany; 2 Kliniken Schmieder Heidelberg, Heidelberg, Germany; 3 Neurologische Klinik Westend, Bad Wildungen, Germany; 4 Asklepios Schlossberg Klinik Bad König, Bad König, Germany; 5 Brandenburg Klinik Bernau, Berlin, Germany; 6 Neurologisches Rehabilitationszentrum “Godeshöhe” Bonn, Bonn, Germany; 7 Neurologische Klinik GmbH Bad Neustadt, Bad Neustadt an der Saale, Germany; 8 Schön Klinik Bad Aibling, Bad Aibling, Germany; 9 Helios Klinik Kipfenberg, Kipfenberg, Germany; 10 BDH-Klinik Greifswald, Greifswald, Germany; 11 Hegau-Jugendwerk Gailingen, Gailingen, Germany; 12 Asklepios Kliniken Schildautal Seesen, Seesen, Germany; 13 Schön Klinik Bad Staffelstein, Bad Staffelstein, Germany; 14 BDH-Klinik Elzach, Elzach, Germany; 15 Neurologisches Rehabilitationszentrum Leipzig, Bennewitz, Germany; 16 Department of Public Health, University of Dresden, Dresden, Germany; 17 Department of Public Health, University of Dresden, Dresden, Germany; 18 Klinik Schloss Pulsnitz, Pulsnitz, Germany

**Keywords:** MRSA, ESBL, Early rehabilitation, Outcome

## Abstract

**Background:**

Colonization or infection with multi-drug resistant (MDR) bacteria is considered detrimental to the outcome of neurological and neurosurgical early rehabilitation patients.

**Methods:**

In a German multi-center study, 754 neurological early rehabilitation patients were enrolled and and reviewed in respect to MDR status, length of stay (LOS) and the following outcome variables: Barthel Index (BI), Early Rehabilitation Index (ERI), Glasgow Outcome Score Extended (GOSE), Coma Remission Scale (CRS), Functional Ambulation Categories (FAC).

**Results:**

The mean age of the study population was 68.0 ± 14.8 years. Upon admission, the following prevalence for MDRs was observed: MRSA (methicillin resistant staphylococcus aureus) 7.0% (53/754), ESBL- (extended spectrum beta-lactamase) producing bacteria strains 12.6% (95/754), VRE (vancomycin resistant enterococci) 2.8% (21/754). Patients colonized or infected with MDR bacteria (MDR+) were significantly more frequently diagnosed with a critical illness polyneuropathy – CIP – than non-colonized (MDR-) patients: 29.0% vs. 14.8%. In addition, they were more frequently mechanically ventilated (MDR+: 55/138, 39.9%; MDR- 137/616, 22.2%). MDR+ patients were referred to rehabilitation earlier, had a longer LOS in early rehabilitation, lower BI on admission and at discharge, lower ERI on admission and lower CRS at discharge than MDR- patients. There was a highly significant correlation of the BI upon admission with the BI at discharge (r_s_ = 0.492, *p* < 0.001). GOSE at discharge differed significantly between both groups (*χ* ^2^-test, *p* < 0.01). Perhaps of greatest importance, mortality among MDR+ was higher in comparison to MDR- (18.1% vs. 7.6%).

**Conclusions:**

The outcome of neurological early rehabilitation patients colonized or infected with MDR bacteria including MRSA or ESBL producing strains is significantly poorer than by non-colonized patients. There is some evidence that the poor outcome could be related to the higher morbidity and lower functional status upon admission.

## Background

In Germany, there exist an increasing number of specialized hospitals for early neurological and neurosurgical rehabilitation. These hospitals continue intensive and intermediate care treatment of patients who have been treated in intensive care units of acute-care facilities, who do not require specialized interventions but are still dependent on an intensive care setting. Many patients cared for in neurological and neurosurgical early rehabilitation facilities were previously treated in non-neurological/non-neurosurgical clinics, most of them suffering from neurological complications including critical illness polyneuropathy (CIP), hypoxic or septic encephalopathy [1].

Colonization and/or infection with multi-drug resistant (MDR) bacteria including methicillin resistant Staphylococcus aureus (MRSA) or extended spectrum beta-lactamase (ESBL) producing gram-negative strains is a challenge in neurological and neurosurgical early rehabilitation. To date, it has been shown that MRSA prevalence upon admission ranges from 7.0 to 14.5% [1–3], whereas colonization with ESBL producing bacteria from 12.6 to 14.0% [1, 3, 4]. Other MDR strains including vancomycin-resistant enterococci (VRE) play a minor role, being present in only approximately 2.8% of cases [1].

This means that almost every fourth patient entering neurological early rehabilitation is either colonized or infected with multi-drug resistant bacteria [1–4].

When a patient with MRSA or ESBL-producing bacteria is admitted, contact precautions (isolation) are recommended in Germany, in particular on intensive or intermediate care units [5, 6]. Obviously, isolation measures in rehabilitation facilities raise ethical concerns [7], due to their being responsible for symptoms of psychological distress including depression and anxiety [8]. Isolation measures interfere with the traditional rehabilitative approach and may cause poorer outcomes [7].

The impact of contact precautions on the outcome of neurological early rehabilitation patients was recently investigated in two single-center studies [9, 10]. It was shown that functional recovery of patients colonized or infected with either MRSA or ESBL-producing bacteria was poorer than in non-colonized patients [9, 10]. However, the poorer outcome was not a result of less therapy (due to isolation) but due to the lower functional status and higher morbidity upon admission [9, 10].

The present paper is based upon data from a 2014 German multi-center study on neurological and neurosurgical early rehabilitation [1]. The focus of the present investigation was to analyze the influence of multi-drug resistant bacteria upon the outcome of the rehabilitation measure.

## Methods

The multi-center study collected data prospectively from 754 patients admitted to 16 German neurological early rehabilitation centers in March, 2014 [1]. Prevalence of patients carrying MDR bacteria (colonization or infection) on admission was documented.

Age, gender, primary diagnosis, medical devices, Glasgow Coma Scale (GCS) [11], Coma Remission Scale (CRS) [12], Barthel Index (BI) [13], Early Rehabilitation Index (ERI) [14] and FAC (Functional Ambulation Categories) [15] on admission and at discharge were documented. In addition, Glasgow Outcome Scale-Extended (GOSE) [16] was obtained at discharge.

Statistics: Since main variables such as BI on admission and at discharge were not normally distributed, non-parametric testing was performed. Statistical analyses included *χ* ^2^-, Mann-Whitney-U-tests for independent samples and Spearman-Rho correlations. Differences were regarded as significant with *p* < 0.05. Nonetheless, in the results section, mean values and standard deviations are displayed.

## Results

The mean age of the whole study sample was 68.0 ± 14.8 years; 297 (39.4%) subjects were female. On admission, MRSA prevalence was 7.0% (53/754), ESBL-producing bacteria were present in 12.6% (95/754), while VRE were found in only 2.8% (21/754) of cases. Carriers of MRSA, ESBL-producing germs or VRE were assigned to the multi-drug resistant positive (MDR+) group (138/754, 18.3%), all other patients were regarded as MDR negative (MDR-). 31 MDR+ patients were colonized with more than one MDR strain.

Primary diagnoses and characteristics of MDR+ and MDR- patients are to be found in Tables 1 and 2.

**Table 1:** Primary diagnoses of early rehabilitation patients with (MDR+) and without (MDR-) multi-drug resistant (MDR) bacteria

	MDR+	MDR-	Sum
Polyneuropathy or other peripheral nerve impairment	40 (29.0%)	91 (14.8%)	131
Ischemic stroke	32 (23.2%)	207 (33.6%)	239
Intracranial hemorrhage	22 (15.9%)	132 (21.4%)	154
Traumatic brain injury	14 (10.1%)	73 (11.9%)	87
Hypoxia	9 (6.5%)	38 (6.2%)	47
Spinal cord injury	9 (6.5%)	19 (3.1%)	28
Brain tumor	3 (2.2%)	18 (2.9%)	21
Other primary diagnosis	9 (6.5%)	38 (6.2%)	47
Sum	138 (100%)	616 (100%)	754

**Table 2:** Characteristics of MDR+ and MDR- neurological early rehabilitation patients

	MDR+	MDR-	*p*-value*
N	138	616	-
Sex (m/w)	90/48	367/249	n.s.**
Age [years]	67.9 (13.7)	68.0 (15.0)	n.s.
Disease onset [days prior to admission]	51.5 (59.9)	75.1 (557.8)	<0.001
Length of stay in neurological early rehabilitation [days]	69.0 (64.7)	54.3 (46.3)	<0.01
Barthel Index (BI) on admission [0 to 100]	4.7 (7.3)	9.3 (11.9)	<0.001
BI at discharge [0 to 100]	20.0 (22.4)	26.2 (22.8)	<0.01
Λ BI (discharge-admission)	15.3 (21.5)	16.8 (19.5)	n.s.
Early Rehabilitation Index (ERI) on admission [−325 to 0]	−129.0 (86.6)	−108.8 (84.3)	<0.05
ERI at discharge [−325 to 0]	−62.5 (75.4)	−53.5 (63.8)	n.s.
Λ ERI (discharge-admission)	55.3 (74.0)	66.5 (82.5)	n.s.
Coma Remission Scale (CRS) on admission [0 to 24]	17.8 (7.8)	18.4 (7.4)	n.s.
CRS at discharge [0 to 24]	18.1 (9.2)	20.3 (7.4)	<0.05
Λ CRS (discharge-admission)	0.0 (9.5)	1.6 (7.3)	n.s.
Glasgow Coma Scale (GCS) on admission [3 to 15]	11.5 (3.8)	11.8 (3.6)	n.s.
GCS at discharge [3 to 15]	11.7 (4.7)	12.9 (3.6)	n.s.
Λ GCS (discharge-admission)	0.1 (4.9)	1.1 (3.4)	n.s.

Mean and standard deviation are displayed

* Mann-Whitney-*U*-test, ** *χ* ^2^-test, n.s. = not significant (*p* > 0.05)

Diagnoses were unequally distributed between MDR+ and MDR- (χ^2^-test, *p* < 0.001), Table 1. The most frequent diagnosis among MDR+ was critical-illness polyneuropathy (CIP) and a higher proportion of MDR+ patients suffered from CIP compared to MDR- (29.0% vs. 14.8%). This finding could be explained by the fact that relatively more MDR+ were mechanically ventilated on admission (MDR+: 55/138, 39.9%; MDR- 137/616, 22.2%) and CIP was the leading diagnosis among ventilated patients (71/192, 37.0%). Ventilated patients had a significantly lower BI on admission (*p* < 0.001) and at discharge (*p* < 0.001) than spontaneously breathing patients.

MDR+ patients were referred to rehabilitation earlier, had a longer length of stay (LOS) in early rehabilitation, lower BI on admission and at discharge, lower ERI on admission and lower CRS at discharge, Table 2. BI upon admission correlated significantly with BI at discharge (r_s_ = 0.492, *p* < 0.001). The older the patients, the lower the BI at discharge was documented (r_s_ = −0.174, *p* < 0.001). However, MDR+ and MDR- did not differ significantly with respect to age (Table 2).

GOSE at discharge differed significantly between both groups (χ^2^-test, *p* < 0.01), in particular mortality among MDR+ was higher compared to MDR- (18.1% vs. 7.6%), Table 3.

**Table 3:** Glasgow Outcome Scale - Extended (GOSE) at discharge

GOSE	MDR+	MDR-	Sum
1 death	25 (18.1%)	47 (7.6%)	72 (9.5%)
2 vegetative state	12 (8.7%)	45 (7.3%)	57 (7.6%)
3 lower severe disability	41 (29.7%)	246 (39.9%)	287 (38.1%)
4 upper severe disability	58 (42.0%)	257 (41.7%)	315 (41.8%)
5 lower moderate disability	2 (1.4%)	21 (3.4%)	23 (3.1%)
6 upper moderate disability	0	0	0
7 lower good recovery	0	0	0
8 upper good recovery	0	0	0
Sum	138 (100%)	616 (100%)	754 (100%)

Neither FAC on admission (Table 4) nor at discharge (Table 5) differed significantly between both groups (χ^2^-test, n.s.).

**Table 4:** Functional Ambulation Categories (FAC) upon admission

FAC	MDR+	MDR-	Sum
0 - patient cannot walk	128 (92.8%)	541 (88.1%)	669 (89.0%)
1 - patients needs firm continuous support from 1 person who helps carrying weight and with balance	6 (4.3%)	45 (7.3%)	51 (6.8%)
2 - patient needs continuous or intermittent support of one person to help with balance and coordination.	2 (1.4%)	18 (2.9%)	20 (2.7%)
3 - patient requires verbal supervision or stand-by help from one person without physical contact	2 (1.4%)	9 (1.5%)	11 (1.5%)
4 - patient can walk independently on level ground, but requires help on stairs, slopes or uneven surfaces	0	1 (0.2%)	1 (0.1%)
5 - patient can walk independently anywhere	0	0	0
Sum	138 (100%)	614 (100%)	752 (100%)

**Table 5:** Functional Ambulation Categories (FAC) at discharge

FAC	MDR+	MDR-	Sum
0 - patient cannot walk	88 (65.2%)	336 (56.0%)	424 (57.7%)
1 - patients needs firm continuous support from 1 person who helps carrying weight and with balance	16 (11.9%)	69 (11.5%)	85 (11.6%)
2 - patient needs continuous or intermittent support of one person to help with balance and coordination.	12 (8.9%)	85 (14.2%)	97 (13.2%)
3 - patient requires verbal supervision or stand-by help from one person without physical contact	10 (7.4%)	64 (10.7%)	74 (10.1%)
4 - patient can walk independently on level ground, but requires help on stairs, slopes or uneven surfaces	8 (5.9%)	37 (6.2%)	45 (6.1%)
5 - patient can walk independently anywhere	1 (0.7%)	9 (1.5%)	10 (1.4%)
Sum	135 (100%)	600 (100%)	735 (100%)

## Discussion

The high prevalence of MDR bacteria is an emerging challenge in neurological and neurosurgical early rehabilitation. In the present study, 18.3% of all patients were either colonized or infected with MDR bacteria upon admission. While MRSA prevalence (7.0%) was lower than reported before [2, 3], colonization with MDR gram negative strains (12.6%) was in line with previous studies [2, 4]. MRSA and ESBL producing bacteria proved to be quite common, whereas the VRE prevalence was less than 3%.

It has previously been shown in a single-center study that patients with MRSA or ESBL-producing germs are at risk for a poor outcome [9, 10]. The present multi-center data support this hypothesis. MDR+ had a significantly worse functional status – as measured with the BI – at discharge. While FAC did not show significant differences between MDR+ and MDR-, the GOS was poorer among carriers of MDR bacteria.

How are these data to be interpreted? First of all, functional status on admission was lower among MDR+ patients. Some authors have suggested that low a functional status upon admission may be one of the most important predictors for a poor outcome [9, 10]. There was a clear, highly significant correlation between BI on admission and at discharge in the present study sample. Furthermore, ERI on admission was lower in the MDR+ group. The ERI provides a good estimate of early rehabilitation patients’ morbidity [14, 17]. In addition, we found that among patients on mechanical ventilation, a significantly higher proportion of patients were MDR+. It is well known that mechanically ventilated patients have a higher morbidity, mortality and poorer outcome in neurological early rehabilitation [1, 18, 19].

In addition, improvements in the assessments (ERI, CRS and GCS) from admission to discharge were not differing between MDR+ and MDR- groups. As far as the changes in BI (discharge minus admission) are concerned, it has to be pointed out that there were no significant differences between the groups, either. So, MDR+ patients benefitted from neurological early rehabilitation, despite lower functional status on admission and contact precautions.

Another finding was that MDR+ patients were referred earlier to rehabilitation from acute-care hospitals than MDR-. One possible explanation for this finding is the fact that colonization with MDR bacteria pose a significant economic burden, since patients colonized with MRSA and ESBL producing bacteria have to be frequently isolated, thus occupying hospital beds [3]. This might contribute to earlier transfers to rehabilitation facilities in order to minimize financial losses in acute-care hospitals. In addition, patients requiring prolonged weaning from mechanical ventilation are steadily increasing in early rehabilitation facilities [1, 18, 19]. Overall, this is a welcome trend since neurologically impaired patients, e.g., following stroke, may profit from an early transfer [20]. Further studies are required on the impact of MDR bacteria on the outcome of neurological and neurosurgical early rehabilitation patients.

## Conclusions

The outcome of neurological early rehabilitation patients with multi-drug resistant (MDR) bacteria such as MRSA or ESBL producing strains is poorer than by non-colonized patients. There is some evidence that the poor outcome may be explained by higher morbidity and lower functional status on admission.

## Abbreviations

BI: Barthel Index
CIP: Critical illness polyneuropathy
CRS: Coma remission scale
ERI: Early rehabilitation index
ESBL: Extended spectrum beta-lactamase
FAC: Functional ambulation categories
GCS: Glasgow coma scale
GOSE: Glasgow outcome scale extended
LOS: Length of stay
MDR: Multi-drug resistant
MRSA: Methicillin resistant staphylococcus aureus
VRE: Vancomycin resistant enterococci
